# Overnight dynamics in scale-free and oscillatory spectral parameters of NREM sleep EEG

**DOI:** 10.1038/s41598-022-23033-y

**Published:** 2022-11-01

**Authors:** Csenge G. Horváth, Orsolya Szalárdy, Péter P. Ujma, Péter Simor, Ferenc Gombos, Ilona Kovács, Martin Dresler, Róbert Bódizs

**Affiliations:** 1 Institute of Behavioural Sciences, Semmelweis University, Budapest, Hungary; 2 Institute of Cognitive Neuroscience and Psychology, Research Centre for Natural Sciences, Budapest, Hungary; 3 Institute of Psychology, ELTE, Eötvös Loránd University, Budapest, Hungary; 4 UR2NF, Neuropsychology and Functional Neuroimaging Research Unit at CRCN-Center for Research in Cognition and Neurosciences and UNI-ULB Neurosciences Institute, Université Libre de Bruxelles (ULB), Brussels, Belgium; 5 Laboratory for Psychological Research, Pázmány Péter Catholic University, Budapest, Hungary; 6 ELRN-ELTE-PPKE Adolescent Development Research Group, Faculty of Education and Psychology, Eötvös Loránd University, Budapest, Hungary; 7 Donders Institute for Brain, Cognition and Behaviour, Radboud University Medical Center, Nijmegen, The Netherlands

**Keywords:** Circadian regulation, Non-REM sleep, Sleep

## Abstract

Unfolding the overnight dynamics in human sleep features plays a pivotal role in understanding sleep regulation. Studies revealed the complex reorganization of the frequency composition of sleep electroencephalogram (EEG) during the course of sleep, however the scale-free and the oscillatory measures remained undistinguished and improperly characterized before. By focusing on the first four non-rapid eye movement (NREM) periods of night sleep records of 251 healthy human subjects (4–69 years), here we reveal the flattening of spectral slopes and decrease in several measures of the spectral intercepts during consecutive sleep cycles. Slopes and intercepts are significant predictors of slow wave activity (SWA), the gold standard measure of sleep intensity. The overnight increase in spectral peak sizes (amplitudes relative to scale-free spectra) in the broad sigma range is paralleled by a U-shaped time course of peak frequencies in frontopolar regions. Although, the set of spectral indices analyzed herein reproduce known age- and sex-effects, the interindividual variability in spectral slope steepness is lower as compared to the variability in SWA. Findings indicate that distinct scale-free and oscillatory measures of sleep EEG could provide composite measures of sleep dynamics with low redundancy, potentially affording new insights into sleep regulatory processes in future studies.

## Introduction

The dynamics of NREM sleep EEG spectral features over consecutive sleep cycles is of crucial importance in understanding the regulation and the function of sleep^1,2^, as well as in revealing the physiological bases of insomnia^3^ and major depressive disorder^4^. The well-known physiological marker of sleep intensity, the slow wave activity (SWA, power in the 0.75–4.5 Hz range) of sleep EEG was shown to decrease exponentially in consecutive sleep cycles^5,6^ reflecting overall sleep–wake history^7^. Furthermore, much less emphasized observations suggest that in contrast to SWA, the power in several spectral bins of the sleep spindle frequency range increases across sleep cycles, whereas beta activity remains nearly stable over consecutive NREM periods^1^. In addition, the time course of gamma EEG activity is characterized by slight overnight increase^8^, whereas both beta and gamma frequency EEG activity in NREM sleep are attenuated after periods of extended wakefulness^9^. Last, but not least, both the occurrence rates and the amplitudes of slow and fast sleep spindles were characterized by changing dominance (overnight decreases and increases for slow and fast sleep spindles, respectively), whereas frequencies were shown to follow a U-shaped overnight distribution over consecutive sleep cycles, with decelerations of 0.1 Hz during the middle part of the sleep period for both sleep spindle types^10^. These findings indicate that the overnight dynamics of NREM sleep EEG is a complex and multi-faceted process, involving a broad range of frequencies of the spectrum, but these constituents are not yet fully unraveled at the current stage of knowledge in the field.

Prior research generally confirms that neurophysiological signals comprise a rhythmic oscillatory-, and an arrhythmic (or aperiodic) activity. Thanks to its special statistical property the scalp electroencephalogram (EEG) spectrum follows a power-law distribution^11,12^, thus, there is a linear relationship between the logarithm of amplitude and the logarithm of frequency^13^. This 1/f relationship is the aperiodic component of the signal due to the self-evident scale-free being of the power-law functions. Namely, such a scale-invariant nature suggests that no specific frequency dominates the signal, rather, the spectral slope reflects the overall frequency composition within the time series. The oscillatory part of the power spectrum is shown as upward deflections in specific frequency bands.

Analysing pre-defined oscillatory bands contains several possibilities for biases due to the so-called “researcher degrees of freedom”^14^. That is why the parametrization of neural power spectra has gained much importance in recent years^15^. Former reports on the aperiodic, 1/f-type measures of EEG suggest that depth of sleep or sleep intensity could indeed be reflected by the spectral slope (or exponent) of the signal. Findings which support this assumption were reported in studies revealing the sensitivity of EEG and electrocorticogram (ECoG) spectral slopes in discriminating wakefulness from states of reduced arousal including NREM and REM sleep, as well as general anesthesia or unconsciousness^16,17^. Furthermore, increasingly negatively sloped EEG and ECoG power spectra were reported from wakefulness through REM and N2 to N3 sleep states^11,18,19^. Last, but not least modelling studies indicate that spectral slopes of neural time series data reflect the ratio of inhibition over excitation in the underlying neural tissue, where increased inhibition associates with steeper slopes^20^. However, none of the above studies explicitly focused on across sleep cycle (or overnight) dynamics of spectral slopes, which is of crucial importance in depicting the regulatory aspects of sleep. Moreover, no former study analyzing 1/f-type activity characterized the dynamics of other parameters of the NREM sleep EEG spectra, namely the intercept (or amplitude multiplier) and the parameters of the major spectral peaks (peak power and peak frequency). Here we suggest that an appropriate separation of the rhythmic and aperiodic components of non-rapid eye movement (NREM) sleep EEG activity may provide feasible and non-redundant indicators of known across sleep cycle dynamics hypothetically linked to sleep regulatory processes.

Moreover, it was found that interindividual differences in slow wave sleep (SWS) and in quantitatively evaluated delta power in the NREM sleep are considerable, exceeding the effect sizes attributable to sleep–wake history^21,22^. The striking interindividual variability of healthy subjects lead several researchers to the conclusion that available laboratory measures of sleep are inappropriate for the construction of reference values^23^ or for defining "normal" sleep^24^, reducing the sensitivity and specificity of clinical tests based on sleep parameters^22^. This means that despite its importance in sleep medicine and research, it is nearly impossible to give an exact metric as a standard for the measurement of overnight sleep dynamics and intensity. In order to provide a more comprehensive picture of across sleep cycle dynamics here we test the spectral slope and peak parameters in terms of overnight changes and associations with SWA. The set of parameters analyzed in our report were found to be composite, non-redundant and efficient in characterizing known age- sex- and cognitive correlates of sleep^14^. Here we test the feasibility of describing overnight sleep dynamics by this set of parameters, assuming that we will encounter a lower interindividual variability, hence, opening the way to define reference values for healthy sleep cycle dynamics. In order to facilitate the reliance on reference values we publish detailed descriptive statistics of our dataset, which might help other researchers in circumscribing the range of healthy sleep in future studies.

We used the method from our earlier publication^14^ to obtain the slope and the most prominent peak of the Fourier spectrum, that is, to distinct the periodic and aperiodic part of the NREM sleep EEG activity. In this, we proposed that there is a need to include a peak power function in the power law formula as follows:

1 $$P(f) = C{f}^{\alpha }{P}_{Peak}(f)$$

where, P is power as a function of frequency, P_Peak_ is the peak power at frequency f (P_Peak_ (f) = 1 if there is no peak and larger if there is), C is the intercept (a constant) which expresses the frequency-independent EEG amplitude, and α is the spectral slope (spectral exponent; negative number). The latter was associated with sleep depth and arousal level^17,18^. As in our previous work, we define f_maxPeak_ as the frequency at which P_Peak_ reaches its maximum level.

Based on previously published and above cited findings, our main hypotheses are the following:

- The spectral slope (α) and peak amplitude (the maxima of P_Peak_) increase during successive NREM periods (indicating flattening spectra and increasing oscillatory spindle activities, respectively).
- Spectral slope correlates negatively with SWA, the gold-standard measure of sleep intensity.
- The f_maxPeak_ values (i.e. the frequency of the largest peak in the spindle range) in the frontopolar and parieto-occipital regions are characterized by a U-shaped distribution across consecutive sleep cycles (reflecting the overnight dynamics of slow and fast sleep spindle frequencies, respectively).
- f_maxPeak_ values in the central regions are characterized by a linear increase over the night (reflecting the changing dominance of fast over slow sleep spindles which are mixed in this region).

## Results

The number of full sleep cycles were between 2 and 5 among participants (mean: 4.25). All subjects (N = 251) had at least two, 249 had at least three, 240 had four or more, and 75 had five complete sleep cycles. The last (5th) cycles were not included in the models because they would have reduced the sample size considerably. The main effect of hemisphere (left vs. right) was not significant in either of the models, so it was not investigated further (p > 0.05).

### Goodness-of-fit in the separate sleep cycles

As in our earlier publication^14^, the equidistant log–log plots of the NREM EEG power spectra were fitted with linear functions below 48 Hz, furthermore, these fittings was performed with the exclusion of the 0–2 and the 6–18 Hz range to avoid spectral peaks such as slow oscillation and sleep spindles. The sample mean of slope and intercept values were − 2.71 (SD = 0.28) and 5.53 (SD = 1.11) in cycle 1, − 2.61 (SD = 0.38) and 5.03 (SD = 1.01) in cycle 2, − 2.46 (SD = 0.68) and 4.65 (SD = 0.93) in cycle 3, − 2.398 (SD = 0.65) and 4.46 (SD = 0.88) in cycle 4, respectively. Goodness of fit of the linear model of the equidistant 2–6 and 18–48 Hz spectral data varied in the range of 0.9195–0.9998 in cycle 1, 0.9002–0.9998 in cycle 2, 0.8938–0.9998 in cycle 3, 0.7686–0.9997 in cycle 4 across subjects and EEG recording locations. The square of the Fisher Z-transformed, averaged and back-transformed Pearson correlations between the fitted linear and the spectral data is $\overline{R }$^2^ = 0.9844 (SD = 0.42).

### Dynamics of slope and maximal peak amplitude throughout the night

**Figure 1:**
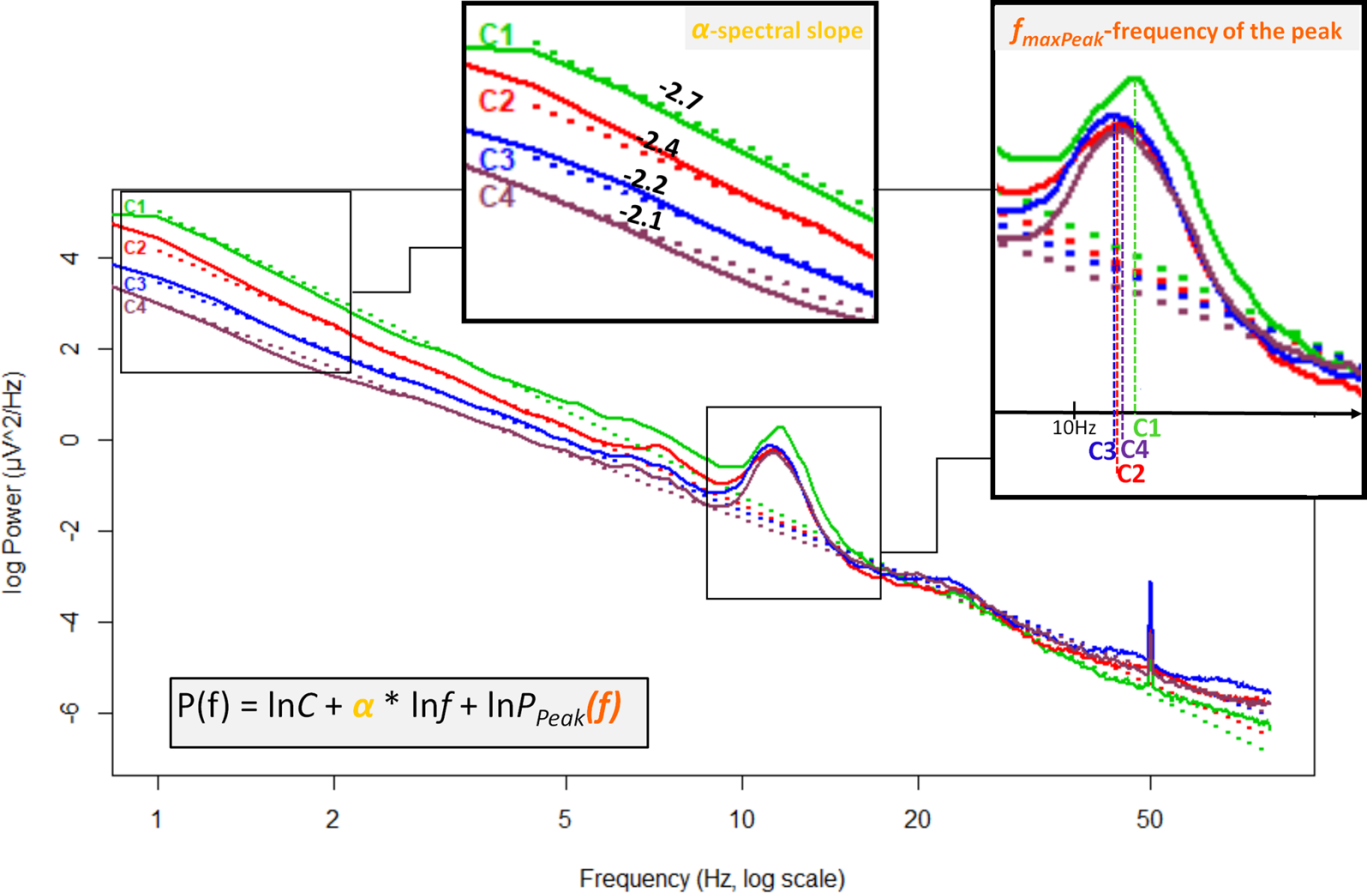
Example for cycle dynamics of spectral slopes and peak frequencies in a 20-year-old male subject in the right frontopolar EEG location (Fp2). C1, C2, C3, C4 reflects cycles 1–4, respectively. Note that slope values show a non-linear increase. Furthermore, the frequencies of the largest peak form a U-shape curve throughout the night, that is, in the middle of the night the peak frequencies are lower than in cycle 1 and 4.

**Figure 2:**
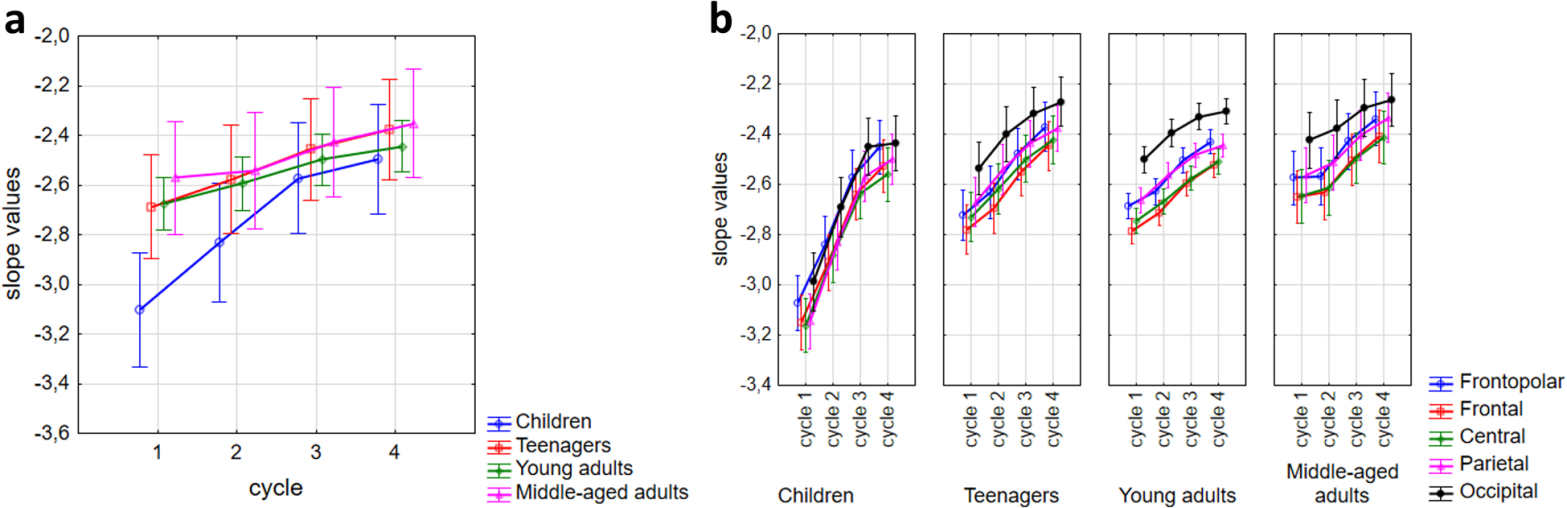
NREM sleep EEG spectral slopes as functions of sleep cycles, age and recording sites. (**a**) Representation of cycle × age group interaction in spectral slope values. (**b**) Depiction of cycle × region × age group interaction in spectral slope values. Dots are group mean values, whereas vertical bars denote 95% confidence intervals.

Sleep cycles (F(3,696) = 210.78, p < 0.001, N = 240), age groups (F(3,232) = 17.05, p < 0.001, N_child_ = 30, N_teenager_ = 36, N_y.adult_ = 142, N_m.a.adult_ = 32; here, and throughout the text, the marks y.adult and m.a.adult refers to young adults and middle-aged adults, respectively) and region (F(4,928) = 211.14, p < 0.001, N = 240) had significant main effects on the slope of the spectrum: later cycles, older ages and more posterior regions predicted smaller absolute values of the spectral slope parameters. In addition these main effects interacted as follows: cycle × age groups (F(9,696) = 15, p < 0.0001), region × age groups (F(12,928) = 3.66, p < 0.0001), cycle × region (F(12,2784) = 51.92, p < 0.0001), indicating accelerated overnight slope changes in children as compared to adults, lack of striking regional differences in children, and regional differences in overnight dynamics, respectively. Post hoc test (see results in Table 2) revealed that slope values were significantly higher (smaller absolute value i.e., flatter slope) in each sleep cycle compared to the preceding ones in the young adult group (see an example in Fig. 1). However, in the children and teenager groups this significantly increasing effect throughout the night was evident just for the first three cycles. Only a trend appeared in the fourth cycles for this effect. Finally, in the middle-aged group, the increment of the spectral slope values was smaller during sleep which resulted in the lack of a significant difference between successive cycles (Fig. 2a). The differences between regions were significant in all cycles (Fig. 2b).

Amplitudes of the largest peaks were significantly affected by age group (F(3) = 11.93, p < 0.001, N_child_ = 9, N_teenager_ = 33, N_y.adult_ = 125, N_m.a.adult_ = 22), cycle (F(3) = 78.45, p < 0.001, N = 189), and region (F(4) = 56.94, p < 0.001, N = 189). Effect of sex was not significant. Cycle × age group (F(9) = 3.65, p < 0.001), region × age group (F(12) = 8.67, p < 0.001), cycle × region (F(12) = 16.64, p < 0.001), indicating attenuated overnight spectral peak amplitude increase in middle-aged adult subjects, decreased peak amplitude in centro-posterior regions of children, faster overnight amplitude increase in posterior regions, respectively. Post hoc test of cycle × age group interaction revealed a significant increase in peak amplitudes throughout the 4 sleep cycles in the young adult group (Table 2).

### Overnight dynamics of SWA and its correlation with the slope

The same models were used to assess SWA at derivation F3. As SWA values are squared numbers, these data evidently do not meet the criteria of parametric statistical tests. Consequently, before running the statistical tests a logarithmic transformation was performed. Nonetheless, the original parameter is the one which is often used in the literature. That is why the general linear model is reported here for both the classical SWA and for the logarithm of SWA (*lnSWA*). However, in the comparative estimations between the slope and SWA the log-transformed metric will be reported.

**Figure 3:**
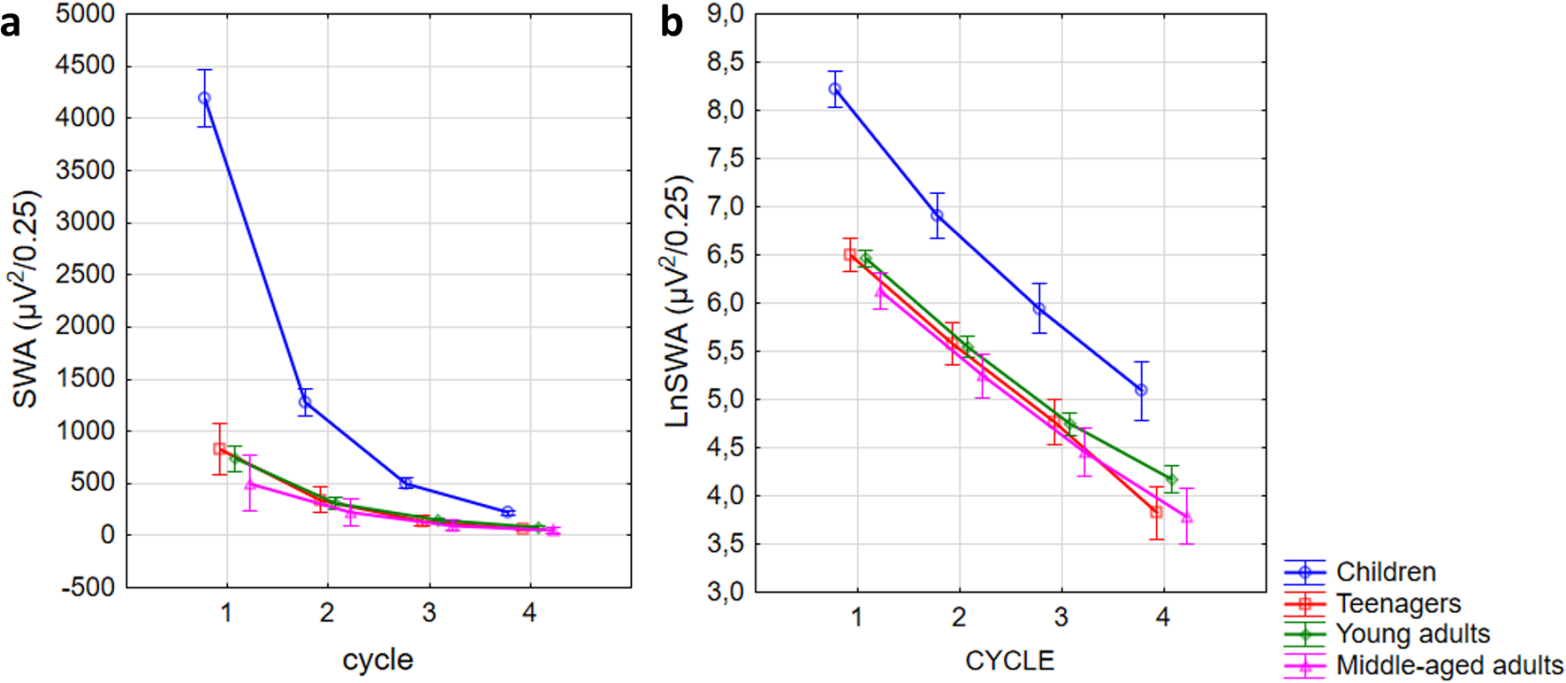
Cycle-by-cycle dynamics of slow wave activity. The left panel shows the common measure of slow wave activity in different age groups and sleep cycles. The natural logarithm of SWA is depicted in the right panel. Note that the decrement of lnSWA is nearly linear.

Cycle (N = 240; SWA: F(3,696) = 524.88, p < 0.001; lnSWA: F(3,696) = 858.84, p < 0.001), and age effect (N_child_ = 30, N_teenager_ = 36, N_y.adult_ = 142, N_m.a.adult_ = 32; SWA: F(232,3) = 707.98, p < 0.001; lnSWA: F(232,3) = 58.68, p < 0.001), in addition, the interaction between them (SWA: F(9,696) = 153.85, p < 0.001; lnSWA: F(9,696) = 5.42, p < 0.001) was evident. Although a significant decrease in logarithmized SWA was evident in all age groups in all cycles according to the post hoc tests (p < 0.001), in case of the classical SWA this effect was only significant in the children and young adult group for the first three (p < 0.01), and in the teenager group for the first two cycles (p < 0.001). In the other cycles and in the middle-aged group the decreasing trend was not significant (p > 0.05) (Fig. 3).

**Figure 4:**
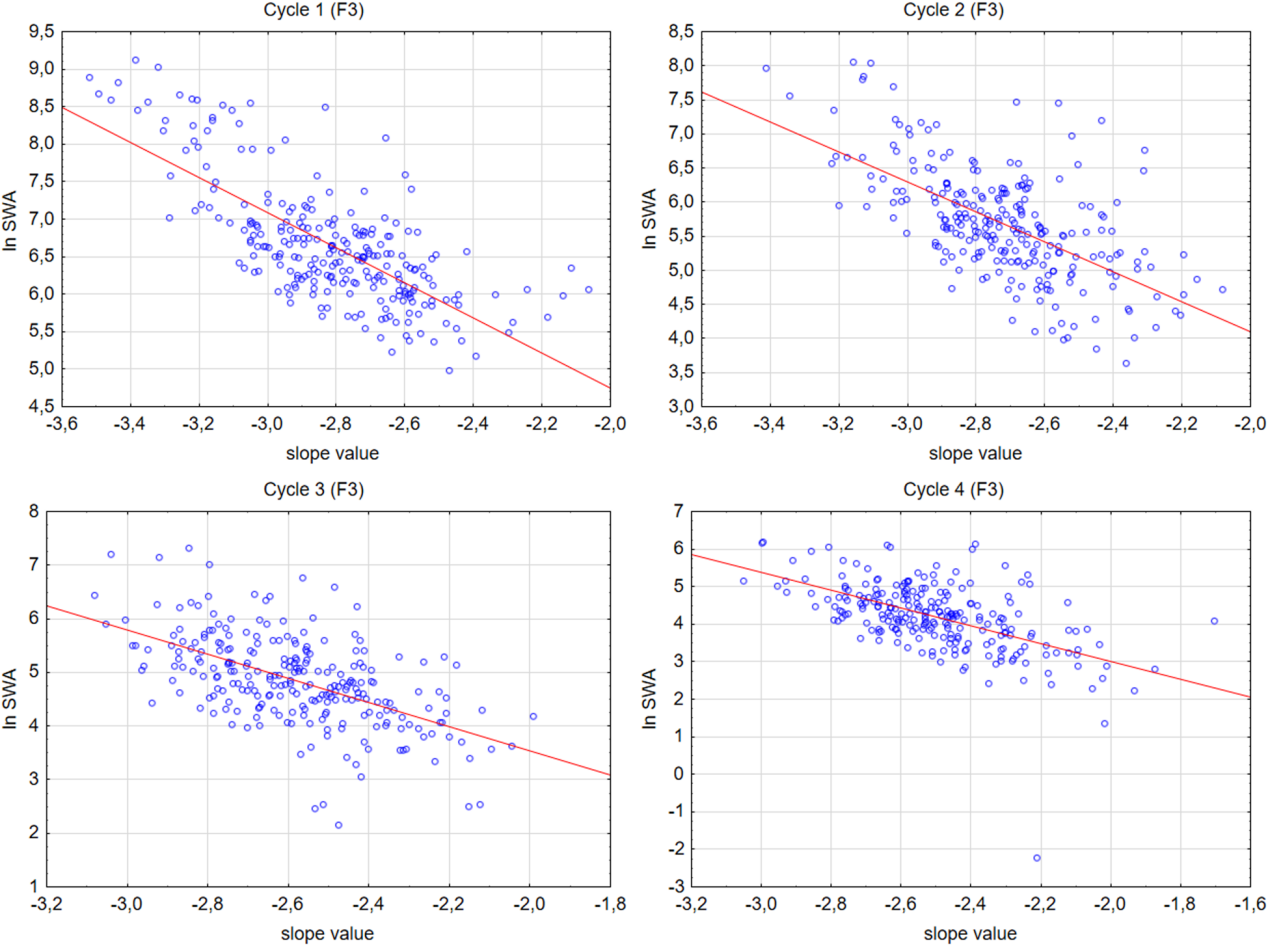
Scatterplots of the correlations between spectral slope value and lnSWA at the left frontal EEG location (F3).

In order to test whether spectral slope values and the natural logarithm of SWA index are overlapping concepts Pearson correlation analyses were applied. Analyses were focused on the left frontal recording location (F3). Significant, negative, moderate to strong correlations were found in all cycles between the two metrics (Fig. 4) (Cycle 1: N = 251 r = − 0.73, p < 0.001; Cycle 2: N = 251 r = − 0.61, p < 0.001; Cycle 3: N = 249 r = − 0.56, p < 0.001; Cycle 4: N = 240 r = − 0.56, p < 0.001).

### Interindividual differences in slope and SWA (/lnSWA) values

**Figure 5:**
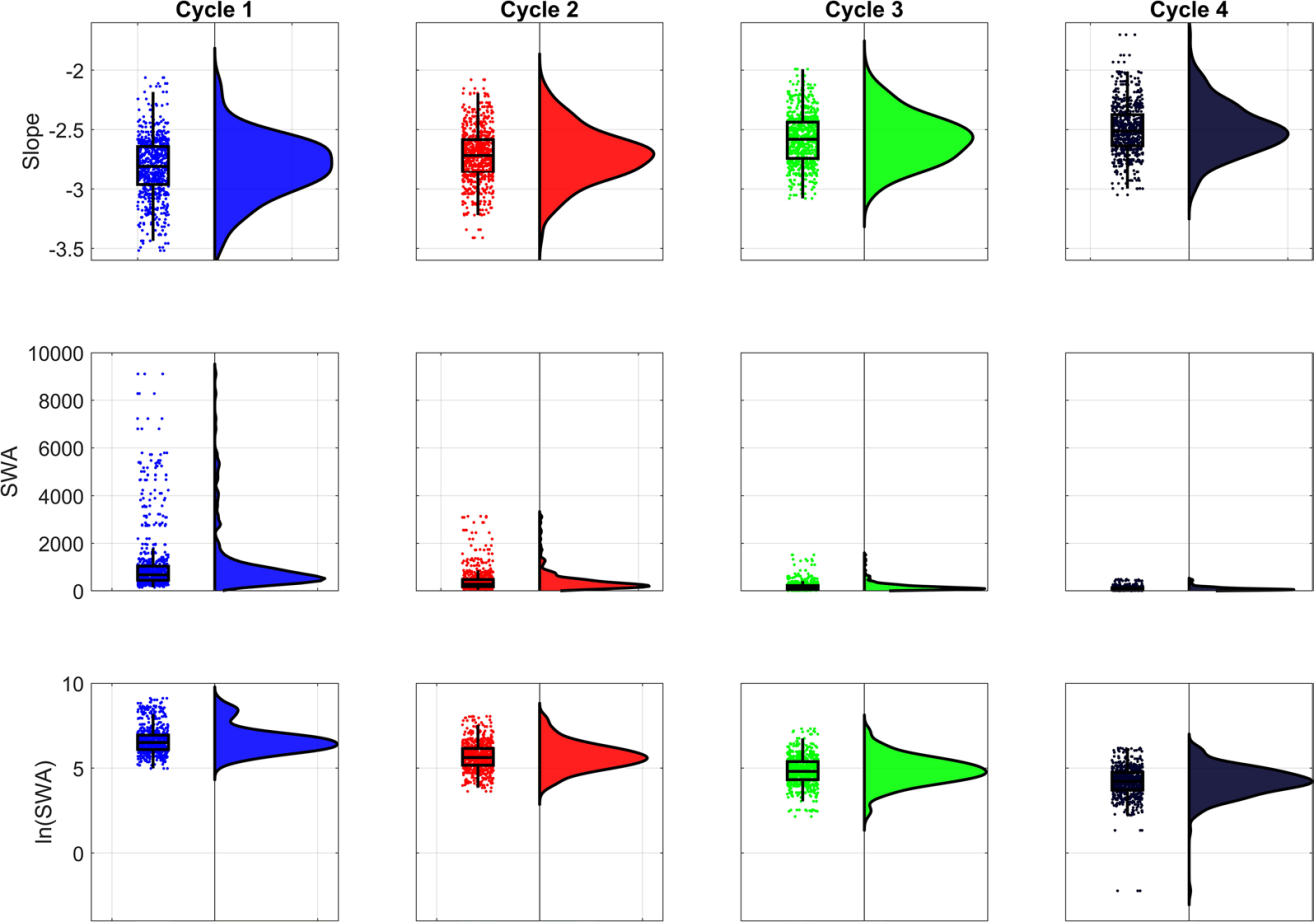
Raincloud plot of NREM sleep EEG spectral slopes, SWA, and lnSWA in the first four sleep cycles. Note the skewness and/or bimodality of the distributions, as well as the increased number of outliers of SWA and lnSWA compared to slope values.

Our main goal was to propose a sleep intensity index which varies less among people than the slow wave activity. Thus, we checked the amount of outliers as well as the coefficients of variation in these two metrics (slope values and SWA) at derivation F3 (Fig. 5).

In order to make the two measures (spectral slopes and lnSWA) comparable there was a need to rescale them according to a common absolute null point, which is a pre-requisite of assessing a relative standard deviation (coefficient of variation). As neither lnSWA nor the slope values were lower than − 4 we add 4 to both variables in all individual datapoints. Based on this rescaling we could calculate the coefficients of variation in both metrics which proved to be more than twice higher in the lnSWA as compared to the spectral slopes (Table 1).

**Table 1:** Descriptive statistics and coefficients of variation of NREM sleep EEG lnSWA and spectral slopes (left frontal recording location: F3).

	Cycle	Valid N	Mean	Minimum	Maximum	Std.Dev.	Coef.Var.
ln SWA+4	1	251	10.65	8.98	13.12	0.81	7.64
	2	251	9.68	7.63	12.05	0.81	8.38
	3	249	8.85	6.15	11.32	0.84	9.46
	4	240	8.18	1.77	10.18	0.91	11.11
slope+4	1	251	6.82	6.06	7.52	0.25	3.72
	2	251	6.72	6.08	7.41	0.23	3.38
	3	249	6.58	5.99	7.08	0.21	3.16
	4	240	6.50	5.70	7.05	0.21	3.30

### Is there a cycle dynamic of the alternative intercepts?

Here we did not analyse the original intercept considering its interdependency with the slope. The square of the Fisher Z-transformed, averaged and back-transformed Pearson correlations between the original intercept (lnC_0_ at ln f = 0, f = 1 Hz) and the spectral slope is: cycle 1: $\overline{R }$^2^ = 0.69 (SD = 0.11), cycle 2: $\overline{R }$^2^ = 0.61 (SD = 0.11), cycle 3: $\overline{R }$^2^ = 0.54 (SD = 0.09), cycle 4: $\overline{R }$^2^ = 0.50 (SD = 0.06). However, cycle significantly affected both alternative intercepts: lnC_2.5_ (F(3,696) = 339.9, p < 0.0001) and lnC_2.6_ (F(3,696) = 274.8, p < 0.0001). Post hoc test of cycle effect revealed a significant difference between all cycles with respect to lnC_2.5_ (N = 240, p < 0.001, M_C1_ = − 1.08, SE = 0.03; M_C2_ = − 1.32, SE = 0.03; M_C3_ = − 1.4, SE = 0.03; M_C4_ = − 1.43, SE = 0.03) as well with regard lnC_2.6_ (N = 240, p < 0.001, M_C1_ = − 1.35, SE = 0.03; M_C2_ = − 1.58, SE = 0.03; M_C3_ = − 1.65, SE = 0.03; M_C4_ = − 1.67, SE = 0.03). Due to the similar cycle dynamics of the two alternative intercepts we hypothesized that the two metrics are highly correlated. The square of the Fisher Z-transformed, averaged and back-transformed Pearson correlations between the two alternative intercepts in the 4 sleep cycles are: cycle 1: $\overline{R }$^2^ = 0.99 (SD = 0.072), cycle 2: $\overline{R }$^2^ = 0.99 (SD = 0.075), cycle 3: $\overline{R }$^2^ = 0.99 (SD = 0.083), cycle 4: $\overline{R }$^2^ = 0.99 (SD = 0.077). Thus we used just lnC_2.5_ for further analyses. In addition to cycle, age groups (F(3,232) = 100.9, p < 0.0001), sex (F(1,232) = 9.6, p < 0.0001) and region (F(4,928) = 110.7, p < 0.0001) had significant main effects on lnC_2.5_. These effects reflect decreasing lnC_2.5_ across successive NREM periods, and age groups, as well as higher lnC_2.5_ in women as compared to men and in anterior as compared to posterior regions. Furthermore, significant interactions were evident for cycle × age groups (F(9,696) = 2.98, p = 0.002), region × age groups (F(12,928) = 4.96, p < 0.0001), region × sex (F(4,928) = 5.51, p = 0.0002), and cycle × region(F(12,2784) = 6.97, p < 0.0001, indicating increased lnC_2.5_ values in first cycle of children, increased lnC_2.5_ in the fronto-central regions of children, decreased lnC_2.5_ in posterior regions of men, lack of decreasing from cycle 3 to 4 in central and parietal regions compared to other regions, respectively.

### Can the classical measure of sleep intensity (SWA) be predicted by the spectral slope and alternative intercept?

As the alternative intercepts have shown a cyclic change, we were curious about how much the slope and the lnC_2.5_ contribute to the nature of SWA in different cycles.

A linear regression model was built to predict SWA from slope of the spectrum and lnC_2.5_ in all cycles (fitted regression model in cycle 1: SWA = − 4293.8–0.44*(slope) + 0.59*(lnC_2.5_); cycle 2: SWA = − 1165.1–0.39*(slope) + 0.59*(lnC_2.5_); cycle 3: SWA = − 378.3–0.36*(slope) + 0.63*(lnC_2.5_); cycle 4: SWA = − 230.6–0.45*(slope) + 0.57*(lnC_2.5_)). The model gave significant result in each case with both of the predictors having a significant contribution (cycle 1: R^2^ = 0.73, F(2,248) = 334.8, p < 0.0001; cycle 2: R^2^ = 0.60, F(2,248) = 187.7, p < 0,0001; cycle 3: R^2^ = 0.58, F(2,246) = 172.9, p < 0,0001; cycle 4: R^2^ = 0.51, F(2,237) = 123.3, p < 0.0001). The contribution of slope was lower than of lnC_2.5_ and it has a negative sign (decrease in lnSWA; cycle 1: β = − 0.44, p < 0.0001; cycle 2: β = − 0.39, p < 0.0001; cycle 3: β = − 0.36, p < 0.0001; cycle 4: β = − 0.45, p < 0.0001) while the alternative intercept contributed to the model with a positive sign (increase in lnSWA; cycle 1: β = 0.59, p < 0.0001; cycle 2: β = 0.59, p < 0.0001; cycle 3: β = 0.63, p < 0.0001; cycle 4: β = 0.57, p < 0.0001). Thus, the two predictors together explained about the 73%, 60%, 58%, 51% of the variance in the 1st, 2nd, 3rd, 4th sleep cycles respectively.

### Overnight change in the frequencies of the maximal peaks (f_maxPeak_) in the spindle frequency range (9–18 Hz)

**Figure 6:**
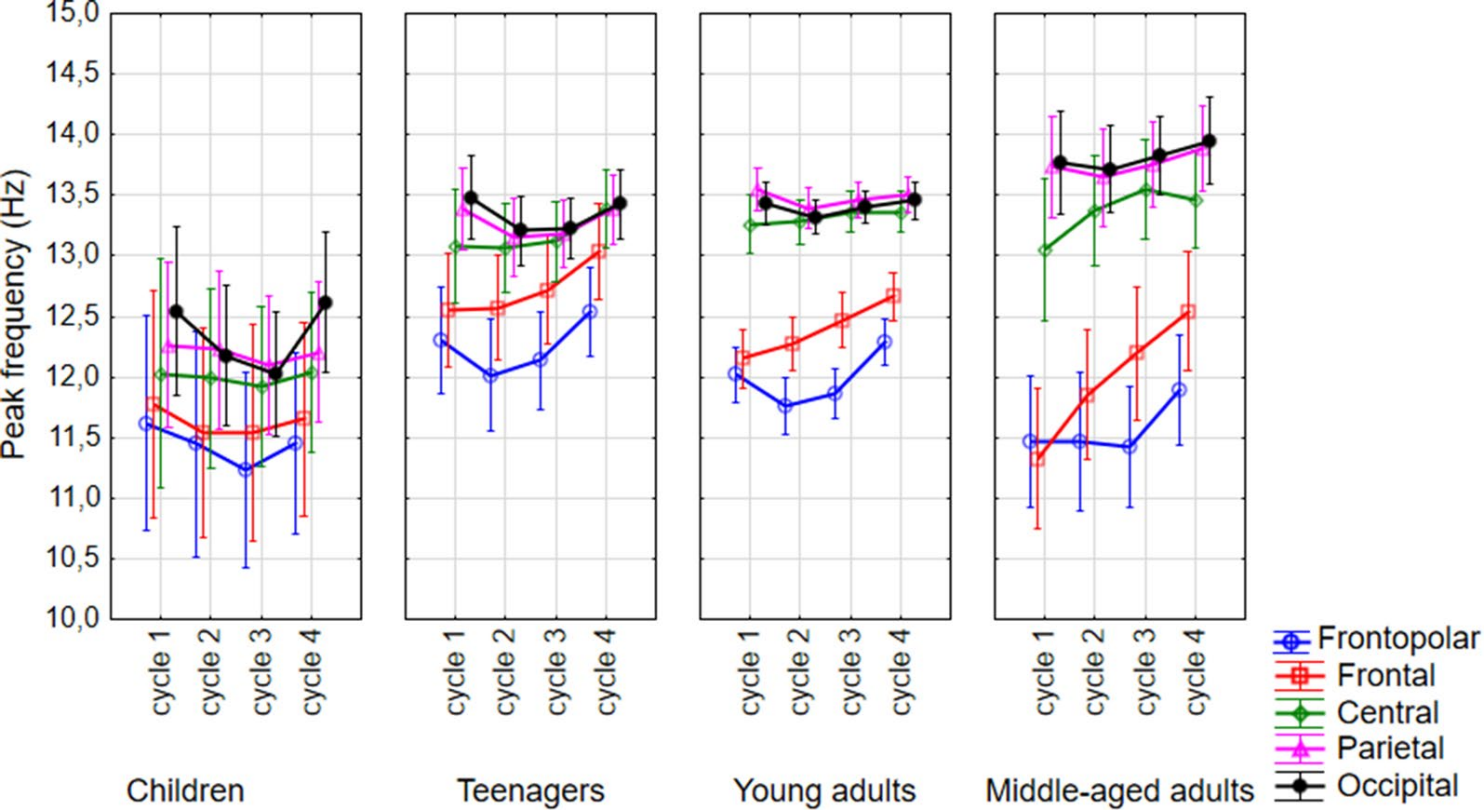
Cycle dynamics of the frequencies of the maximal peaks in the spindle range. Dots are group mean values, whereas vertical bars denote 95% confidence intervals.

Frequencies of the maximal peaks were significantly affected by age (F(3,181) = 10.34, p < 0.001, N_child_ = 9, N_teenager_ = 33, N_y.adult_ = 125, N_m.a.adult_ = 22) sex (F(1,181) = 4.10, p = 0.044, N_male_ = 104, N_female_ = 85), sleep cycles (F(3,543) = 11.65, p < 0.001, N = 189), and region (F(4,724) = 150.10, p < 0.001, N = 189). These effects indicate the already reported lower sleep spindle frequencies in children, adult males, earlier sleep cycles, and more anterior regions, respectively. Although f_maxPeak_ values formed a U-shape-like curve throughout the first 4 sleep cycles (Fig. 6), post hoc test revealed that the first 3 cycles were not significantly different from each other, whereas the f_maxPeak_ values in the 4th cycle were significantly higher than in the others. During the night, the overall frequency increase along the anterior–posterior axis was 1.53 Hz in the 1st, 1.59 Hz in the 2nd, 1.5 Hz in the 3rd, and 1.14 Hz was in the 4th sleep cycle. The maximal value and location of the frequency shifts were also estimated in the different sleep cycles. Antero-posterior frequency shifts in adjacent (para)sagittal regions were tested by an adaptation of the Kullback–Leibler distance type measure involving surrogate control analyses^25^. Tests revealed striking divergences of empirically measured antero-posterior frequency shifts from the uniform distribution indicating largely non-continuous rostro-caudal changes in the frequencies of maximal spectral peaks (Z-values of the Kullback–Leibler distances were 64.69, 84.26, 80.97, and 61.77 for cycles 1, 2, 3, and 4, respectively, p < 0.00001 for each cycle). In terms of descriptive statistics, we found that the maximal frequency shift has a dominant location in every cycle. In the first, second and fourth sleep cycles the central to frontal region was the location of the maximal frequency shift for most of the sample (54.2%, 50.6%, and 40.6%, respectively), whereas in the third cycle, the 45% of the subjects had their maximal frequency shift in the frontal to frontopolar region (Suppl. Table 1).

Significant interaction between sleep cycles × age groups was observed (F(9,543) = 2.38, p = 0.012). Post hoc test of cycle and age groups interaction revealed that f_maxPeak_ values in the fourth cycles of teenagers and young adults are higher than in the previous ones (Table 2). There was a trend for a U-shaped curve (higher first and fourth cycle, lower middle cycles) for all but the middle-aged adult group (Fig. 6). Effects of sleep cycles on maximal peak frequencies depended on electrode location (cycle × region: F(12,2172) = 5.34, p < 0.001). In the frontopolar regions the maximal peak frequencies in the second and third cycles were significantly lower than in the first and fourth (see also Fig. 1). In the parietal and occipital regions there was a trend for this effect, however, the peak frequencies in the frontal and central area did not show a U-shaped curve throughout the night (Table 2). Region and age groups significantly interacted in predicting spectral peak frequencies (F(12,724) = 9.51, p < 0.0001). That is, children had significantly lower peak frequencies in central, parietal and occipital regions than the other groups, whereas teenagers had higher frontopolar and frontal peak frequency values than middle aged adults in the same regions (Fig. 6; Suppl. Table 2, Suppl. Table 3).

**Table 2:** Descriptive statistics and post-hoc test (unequal N HSD) results on the cycle dynamics of the 3 spectral parameters.

		C1 vs. C2 p-value	C2 vs. C3 p-value	C3 vs. C4 p-value	M_c1_	SE_c1_	M_c2_	SE_c2_	M_c3_	SE_c3_	M_c4_	SE_c4_
Slope	Children	< 0.0001	< 0.0001	0.644	− 3.10	0.037	− 2.83	0.038	− 2.57	0.036	− 2.50	0.036
	Teenagers	0.034	0.012	0.452	− 2.69	0.034	− 2.58	0.035	− 2.46	0.033	− 2.38	0.032
	Young adults	< 0.0001	< 0.0001	0.048	− 2.68	0.017	− 2.59	0.018	− 2.50	0.017	− 2.44	0.016
	Middle-aged	1.000	0.052	0.591	− 2.57	0.036	− 2.54	0.038	− 2.43	0.035	− 2.35	0.035
	Frontopolar	< 0.0001	< 0.0001	< 0.0001	− 2.76	0.017	− 2.67	0.018	− 2.50	0.017	− 2.40	0.017
	Frontal	< 0.0001	< 0.0001	< 0.0001	− 2.84	0.017	− 2.74	0.017	− 2.57	0.016	− 2.48	0.016
	Central	< 0.0001	< 0.0001	< 0.0001	− 2.82	0.017	− 2.70	0.017	− 2.55	0.016	− 2.48	0.017
	Parietal	< 0.0001	< 0.0001	< 0.0001	− 2.76	0.017	− 2.61	0.017	− 2.47	0.016	− 2.41	0.016
	Occipital	< 0.0001	< 0.0001	< 0.0001	− 2.61	0.018	− 2.47	0.018	− 2.35	0.018	− 2.32	0.017
Max peak frequency	Children	1.000	1.000	0.972	12.05	0.213	11.88	0.202	11.77	0.183	11.99	0.190
	Teenagers	0.724	1.000	0.011	12.96	0.106	12.81	0.100	12.88	0.090	13.15	0.094
	Young adults	0.834	0.303	0.010	12.88	0.054	12.81	0.051	12.91	0.046	13.05	0.048
	Middle-aged	0.999	0.981	0.578	12.67	0.131	12.81	0.124	12.95	0.112	13.15	0.116
	Frontopolar	< 0.0001	0.980	< 0.0001	11.85	0.100	11.67	0.110	11.66	0.090	12.04	0.090
	Frontal	0.167	0.001	< 0.0001	11.95	0.110	12.06	0.100	12.23	0.100	12.48	0.090
	Central	1.000	0.940	1.000	12.85	0.110	12.93	0.090	12.99	0.080	13.06	0.080
	Parietal	0.043	1.000	0.930	13.23	0.080	13.11	0.080	13.12	0.070	13.24	0.070
	Occipital	0.109	0.999	0.507	13.30	0.080	13.11	0.070	13.12	0.060	13.36	0.070
Max peak amplitude	Children	0.031	0.137	1.000	0.94	0.15	1.28	0.16	1.49	0.16	1.63	0.16
	Teenagers	< 0.0001	0.073	0.999	1.67	0.07	1.92	0.08	2.07	0.08	2.13	0.08
	Young adults	< 0.0001	< 0.0001	0.001	1.85	0.040	2.04	0.040	2.15	0.040	2.26	0.040
	Middle-aged	1.000	0.973	1.000	1.60	0.090	1.67	0.100	1.76	0.100	1.77	0.100
	Frontopolar	0.991	< 0.0001	< 0.0001	1.37	0.063	1.43	0.065	1.54	0.066	1.66	0.068
	Frontal	< 0.0001	< 0.0001	< 0.0001	1.61	0.056	1.75	0.061	1.88	0.059	1.96	0.060
	Central	< 0.0001	< 0.0001	< 0.0001	1.59	0.053	1.88	0.060	2.06	0.057	2.15	0.057
	Parietal	< 0.0001	< 0.0001	< 0.0001	1.72	0.054	2.04	0.060	2.21	0.060	2.28	0.058
	Occipital	< 0.0001	< 0.0001	< 0.0001	1.29	0.053	1.53	0.060	1.66	0.061	1.69	0.059

The table displays the difference between sleep cycles (C1, C2, C3, C4) in particular age groups and regions.

M, mean; SE, standard error.

## Discussion

A precise description of the overnight dynamics of sleep EEG is essential for a better recognition of sleep characteristics and for understanding the underlying processes. In this proof-of-concept study we focused on overnight dynamics of the parameters that describe the power law scaling of the NREM EEG spectrum based on the analysis of successive sleep cycles. Overall, we found that both aperiodic and periodic components of the Fourier spectra undergo remarkable overnight changes. Namely, the slope values increased (absolute values decreased) and the alternative intercepts decreased during the night, besides, the largest peak of the spectrum was characterized by continuously increasing amplitudes in consecutive NREM episodes, and decreased frequencies in the middle of the night in the frontopolar region (U-shape curve in consecutive sleep cycles). Our findings suggest that sleep intensity can be approached by the scale-free measure of frequency dependent decay rate of EEG power, that is by the spectral slope (in the log–log plane) or spectral exponent (in the linear coordinate system). However, the absolute value of the spectral slope (exponent) is not the only measure characterized by overnight decreasing dynamics, nor in predicting SWA, the gold standard measure of sleep intensity. The alternative intercept measure termed lnC_2.5_ is characterized by similar features. Furthermore, the maximal peak frequencies of the Fourier spectra decelerate in the 2. and 3. sleep cycles in the frontopolar, but not in the frontal and central regions. The latter regions might be characterized by the reorganization of the fast and slow spindles during the night.

The number of studies indicating that the power-law exponent of EEG spectra is a correlate of sleep depth, consciousness, and arousal increases steadily. Reduced conscious awareness (or arousal) was related to steeper spectral slopes in these studies^16–18^. Besides, in our former study^14^, we found that aging was associated with decreased steepness of the whole night NREM sleep EEG Fourier spectra. This finding coheres with the literature indicating decreased SWA^26^ and at the same time increased high frequency EEG activity^27^ in the aged. In the present study, we analyzed the cycle-to-cycle dynamics of spectral exponents, as well as SWA at derivation F3. In coherence with the above-mentioned reports, we found an overall age effect in spectral slopes (exponents), SWA and lnSWA. That is with aging, the absolute values of the NREM sleep EEG spectral slopes got smaller at all derivations and in all sleep cycles, whereas the well-known SWA reduction with the progression of age was also evident. A decrement in stage 3 or slow wave sleep, and in slow wave activity (for an overview see^26^) was often associated with the reduction of sleep depth in the elderly. Further similarities between the spectral slopes and SWA was seen in the cycle dynamics of the metrics. Regarding slope values an increasing trend (absolute values declining) can be seen in all age groups throughout the cycles and the opposite trends (because of the opposite sign) were seen in the cycle dynamics of SWA and lnSWA. Thus, increasing and decreasing spectral exponents and SWA in successive sleep cycle were significant in the whole sample, respectively. However, this cycle effect was not significant neither with respect to slopes nor in terms of SWA in the middle-aged group (Fig. 3, Table 2). That is, the increase of the slope values and decrease of SWA throughout the night was smaller in middle-aged as compared to younger subjects. This finding parallels earlier reports on the reduced decay rate of SWA in aged subjects and can be modelled by the assumed attenuation of sleep efficiency in the elderly^28,29^. Nevertheless, it seems that the slope of the Fourier spectrum behaves similar to the classical SWA. Not only with respect to their non-linear decay as sleep progresses, but also regarding age and their regional differences along the fronto-posterior gradient. In the frontal and central regions, the slope was steeper than in the occipital region. It is known that delta waves have higher values over the frontal areas^30^. Furthermore, the two metrics highly correlated which fact alone suggest a convergent validity of the slope. In the light of former studies on sleep depth and spectral exponents and our findings on the spectral slopes in relation with age, cycle dynamics and regional differences, we can suggest that α can be a promising alternative indicator of sleep intensity. Although the reliability and trait-like nature of SWA is indisputable, the slope also has its own advantages. Firstly, this metric is normally distributed, which makes it “usable” in standard statistical models. Secondly, its variability between individuals is much smaller than the interindividual variability of the SWA, even if the log-normalized value of the SWA is considered. That is, the spectral slope is a less individual specific metric. One of the largest criticism in the literature with regard to SWA is its large interindividual variability which make impossible to set up a given reference point for healthy sleep^22–24^. Although slope values smooth down the individual trait effects of specific to the particular subjects as compared to SWA, yet it does not completely blur the fundamental individual differences (such as age or regional), thus it could provide a road toward setting up reference values of sleep intensity in later studies. If this assumption holds true, in the future, we could make difference between healthy and unhealthy sleep with the help of this metric, but less between two individuals with healthy sleep (which latter is one of the main advantage of the classical power values in general and SWA in particular). Hints for the reliability of this assumption were already published in a report indicating flattened spectral slopes in insomnia and sleep misperception subjects^31^. Of course, this idea needs further investigations including even larger datasets, protocols involving the experimental challenge of the sleep homeostat, and /or, measurements of the same subjects across time to reveal inter- and intraindividual differences more widely.

The overnight dynamics of the “slope-free” alternative intercepts were also tested. Results were similar to the ones reported for the spectral slopes in terms of cycle, age, and region effects. However, we found that these alternative intercepts are not independent from the slope if we parametrize the spectra in separate sleep cycles. Indeed, the regression models revealed that these intercepts and the spectral slopes are reliable predictors of SWA. A social isolation EEG study found reduction in broadband power as a result of isolation^32^, suggesting the neural origin and homeostatic relevance of the spectral intercept. However, for a better understanding of the role of broadband power/spectral intercept, as well as the dynamical change of the assumed slope-free intercept during the successive sleep cycles, future studies should explicitly focus on this phenomenon.

Maximal peak frequency values in the spindle frequency range (9–18 Hz) were hypothesized to show a U-shaped distribution over consecutive sleep cycles in the frontopolar and parieto-occipital regions. In a recent report^10^ we revealed that slow and fast sleep spindle frequencies display a U-shaped overnight dynamic, dampening with age and during daytime-sleep sleep spindles are faster than during the night. While our former study revealing the U-shaped overnight dynamics of sleep spindle frequencies was based on the Individual Adjustment Method (IAM)^10^, here we relied on the peak frequency of the whitened power spectra of NREM sleep EEG. Whitening means the removal of the power law trend of the spectra, a step implemented before peak detection in the current, but not in our former study based on the inflection points of the amplitude spectra of NREM sleep EEG. Further differences between our current spectral parametrization and former IAM-based study find its roots in the robust, overall mean frequencies used in the IAM, which contrast the EEG recording location-specific approach in the present report. Last, but not least, IAM sleep spindle frequencies are categorized into slow and fast instances based on both frequency and topography, whereas this distinction is not inherent to the current approach during which we only focused on the EEG recording-specific maxima of the whitened power. Anyhow, the known sex^33,34^, age^35,36^, and region^37^ effects on sleep spindle frequencies were reproduced by this measure. Namely, women had higher, children had lower maximal peak frequencies than men and other age groups, respectively. Furthermore, maximal peak frequencies were lower in anterior as compared to posterior regions. That is, the frequency of the largest peak is roughly corresponding to the sleep spindle frequency. In the present report we found a significant U-shaped curve dynamic of maximal oscillatory peak frequency in the frontopolar region. However, in the frontal and central regions the maximal peak frequencies did not show U-shaped curve during the night. This phenomenon can be due to the changing predominance of slow and fast spindles in consecutive sleep cycles. Fast spindle frequency activity was found to increase during the night, but at the same time a decrease in slow frequency spindling activity was also reported^37,38^. Indeed, in the present study we found that the amplitude of the largest peaks increased in consecutive sleep cycles. As the regional differences between the fast and slow spindle frequencies are powerful and there is a well-known spindle activity growing during the night, it is possible that we see the blurring of the two types of spindles moving towards the central regions. Indeed, in the first two cycles, the dominant location of the maximal frequency shift was the central-to-frontal area in the sample. For the third cycle, this maximal frequency shift moved forward to the frontal-to frontopolar region. This phenomenon can be due to the increase of the fast spindle dominance. However, although in the 4th cycle the maximal frequency shift was occurred again in the central-to-frontal area in most of the subjects (~ 40% of the sample), the difference between the percentage distribution regarding the most and second most dominant location of the maximal frequency shifts getting smaller as the cycles progressed (see Suppl. Table 1). That is, there is an obvious central-to-frontal predominance of the maximal frequency shift in the first cycle but nearly-equal dominances in the 4th cycle regarding central-to-frontal and frontal-to-frontopolar areas. Thus, we suggest that the cycle dynamic of the maximal peak frequencies is contaminated by nocturnal reorganization of the fast and slow spindles in these regions.

There is a lack of and a desperate need for a set of reliable, standardisable polysomnography markers suitable for the assessment of sleep regulation and quality. Such EEG indicators would reduce the complicatedness and arbitrariness of measuring healthy sleep in the related fields of research and medicine. With the present report, we aimed to describe the overnight dynamics of both periodic and aperiodic components of the NREM EEG power spectra. We would like to draw attention to the possibility that these nocturnal patterns may reveal important information on assumed sleep regulatory processes, thus, the description of them is an essential initial step in this field of research. However, there are some shortcomings of this study which need to be handled in the future. Firstly, the parametrization of the power spectra was only adapted to NREM sleep. It would be interesting to test the development of the spectral slope during the whole sleep in all states. Secondly, the frequency range of the linear fitting (2–48 Hz, excluding the 6–18 Hz frequency range) was chosen arbitrarily which can influence the results. In addition, it is known, that the amount and size of spectral peaks can be variable among people, thus considering only the most prominent spectral peaks (in the spindle range) could bias our results. There is a need for more precise extraction of the spindle frequency values from the parametrized power spectra and the comparison our results with other parametrization methods such as Fitting Oscillations & One-Over-F^15^ or Irregular Resampling Auto-Spectral Analysis^39^. Finally, we think that future studies could reveal the role of these parameters in sleep regulatory mechanisms, for example by comparing them with gold standard sleep regulatory indicators (SWA, melatonin, core body temperature) in specific conditions (such as sleep deprivation, sleep displacement).

## Methods

### Subjects

For this retrospective study, second night records of the already published night-time polysomnography data were used from the Budapest-Munich database^40^ to avoid first night effect. Age ranges of the 251 participants (122 females) were between 4 and 69 years with a mean of 25.13. Maximum of two cups of coffee were allowed before noon but alcohol consumption was restricted. Eight participants were light smokers, for them, smoking was not prohibited. All subjects were free of psychiatric or neurological disorders based on self-reports. Ethics Committee of the Semmelweis University (Budapest, Hungary) or the Medical Faculty of the Ludwig Maximilians University (Munich, Germany) approved the research protocols, and the experiment was implemented in accordance with the Declaration of Helsinki. Every participant (or in case of underage, their parents/guardians) signed an informed consent about their attendance in the study.

### Polysomnography

The polysomnography included EEG derivations at Fp1, Fp2, F3, F4, C3, C4, P3, P4, O1, O2 and re-referenced to mathematically linked mastoids, (all of which were placed according to international 10–20 system), furthermore, electromyography (EMG), electro-oculography (EOG) and electrocardiography (ECG) were also used. The sampling frequency for the EEG was either 249 Hz, 250 Hz or 1024 Hz and the impedances for the electrodes were kept below 8 kΩ. After the scoring of the EEG records a 4-s based artefact removal was happened. Determination of sleep cycles was based on the criteria proposed by Aeschbach and Borbély^1^.

### Power spectral analysis

Artefact-free, 4 s epochs with 2 s overlap were Hanning-tapered, mixed-radix fast Fourier transformed, power spectra derived in μV^2^/0.25 Hz. Furthermore, EEG location-specific average power spectral density of the NREM (N2 and N3) periods of sleep cycles was calculated.

#### Definition of SWA

Slow-wave activity was defined as the power spectral density of 0.75–4.5 Hz EEG activity (sum of the bin power values). Analysis was performed on the left frontal EEG recording location (F3) and averaged in chunks of NREM (N2 and N3) periods of complete sleep cycles.

### Spectrum parametrization

The comprehensive description of the parametrization of the power spectra can be seen in our earlier publication^14^, here, the basic steps are summarized. The log–log scale of NREM (N2 and N3) sleep EEG spectra was interpolated to equidistant bins of the smallest frequency step (piecewise cubic Hermite interpolation, 0.0052 Hz). To estimate the slope of the spectrum a linear function was fitted to the data but the frequency ranges below 2 and between 6 and 18 were excluded to avoid the non-random oscillatory parts. The peak detection was conducted in the 9–18 Hz frequency range, known as broad sigma range, searching for local maxima and minima in mathematical terms. Thus, we used the first and second derivative to define critical points and differentiate maxima and minima, respectively. A spectral peak was detected and accepted if the first derivative was equal 0 and the second derivative was smaller than 0.

### Statistical analysis

The 251 subjects were organized in groups along age as in our earlier report^40^. The age ranges in the resulting four groups were the following: 4 years ≤ children < 10 years, N = 31, 15 females; 10 years ≤ teenagers < 20 years, N = 36, 18 females; 20 years ≤ young adults < 40 years, N = 150, 75 females; and 40 years ≤ middle-aged adults ≤ 69 years, N = 34, 14 females. All statistical analyses were carried out with Statistica 13 software. We used general linear models to test our hypotheses. Age groups and sex were between-subject factors whereas sleep cycle, region (frontopolar, frontal, central, parietal, occipital) and hemisphere (left, right) serve as within-subject factors in the models. Second level interactions of the variables with significant main effects are considered herein. With respect to SWA, a logarithmic transformation was also applied. The values of SWA are squared numbers which do not meet the criteria of parametric statistical tests. Thus, in the comparison with the spectral slope we used the logarithm of the SWA values (lnSWA). Furthermore, as SWA was calculated only on the left frontal EEG location we did not include region and hemisphere as factors in the statistical models where we analysed SWA or lnSWA. In case of significant effects or interactions we used the Unequal N HSD post hoc test for further examinations.

The antero-posterior distributions of spectral peak frequencies were tested by the following procedure. First, we formed parasagittal regions by averaging peak frequency values in frontopolar (Fp1, Fp2), frontal (F3, F4), central (C3, C4), parietal (P3, P4), as well as occipital (O1, O2) recording locations. In the following, the regional means of spectral peak frequencies were serially subtracted in adjacent antero-posterior regions as follows: frontal-frontopolar, central-frontal, parietal-central, occipital-parietal^14^. These successive frequency shifts were subjected to an analysis based on an adaptation of the Kullback–Leibler distance (KL distance)^25^ which is widely used in statistics for estimating the difference between two distributions. The mean frequency shifts were calculated for adjacent locations as 4 bins (frontal-frontopolar, central-frontal, parietal-central, occipital-parietal) and normalized dividing each bin value by the sum over the bins, which defines the distribution P(x). The uniform distribution Q(x) was defined as the equal distribution over the four bins, 0.25. The KL distance were then calculated using the following formula: KL = sum(P(x)*log(P(x)/Q(x))). Specifically, we compared the empirical frequency shifts with the outcomes of a randomization based on 1000 iterations in each of the 4 sleep cycles separately. The observed KL values were then z-standardized to the shuffled values, where normalized z-values directly reflect p-values, Z-values of the Kullback–Leibler distances equal to 1.645 corresponds to the 5% p-value, and larger values reflect p < 0.05. We aimed to test if region-specific differences in peak frequencies form a uniform antero-posterior distribution with an equal probability of antero-posterior changes over all adjacent parasagittal regions (null hypothesis assuming continuous antero-posterior changes in peak frequencies) or the changes are non-continuous (assuming a differentiation between slow and fast types of sleep spindles based on non-continuous antero-posterior shifts). Furthermore, we provided a descriptive analysis on the dominant (maximal) frequency shift in each sleep cycle based on our former approach^14^.

### Ethical statement

We confirm that we have read the Journal’s position on issues involved in ethical publication and affirm that this report is consistent with those guidelines.

## Supplementary Information

Supplementary Tables.

## Data Availability

All data generated or analysed during this study are included in this published article (and its Supplementary Information files). The datasets generated during and/or analysed during the current study are available in the OSF repository, https://osf.io/q37w4/.

## References

[1] Aeschbach D, Borbély AA (1993). All-night dynamics of the human sleep EEG. J. Sleep Res..

[2] Dijk DJ, Hayes B, Czeisler CA (1993). Dynamics of electroencephalographic sleep spindles and slow wave activity in men: Effect of sleep deprivation. Brain Res..

[3] Lunsford-Avery JR, Edinger JD, Krystal AD (2021). Optimizing computation of overnight decline in delta power: Evidence for slower rate of decline in delta power in insomnia patients. Clin. Neurophysiol..

[4] Nissen C (2001). Delta sleep ratio as a predictor of sleep deprivation response in major depression. J. Psychiatr. Res..

[5] Dijk DJ (2009). Regulation and functional correlates of slow wave sleep. J. Clin. Sleep Med..

[6] Achermann P, Dijk DJ, Brunner DP, Borbély AA (1993). A model of human sleep homeostasis based on EEG slow-wave activity: Quantitative comparison of data and simulations. Brain Res. Bull..

[7] Dijk DJ, Brunner DP, Beersma DGM, Borbely AA (1990). Electroencephalogram power density and slow wave sleep as a function of prior waking and circadian phase. Sleep.

[8] Ferri R, Elia M, Musumeci SA, Pettinato S (2000). The time course of high-frequency bands (15–45 Hz) in all-night spectral analysis of sleep EEG. Clin. Neurophysiol..

[9] Finelli LA, Ackermann P, Borbély AA (2001). Individual ‘fingerprints’ in human sleep EEG topography. Neuropsychopharmacology.

[10] Bódizs R (2021). Sleep-spindle frequency: Overnight dynamics, afternoon nap effects, and possible circadian modulation. J. Sleep Res..

[11] Pereda E, Gamundi A, Rial R, González J (1998). Non-linear behaviour of human EEG: Fractal exponent versus correlation dimension in awake and sleep stages. Neurosci. Lett..

[12] Pritchard WS (1992). The brain in fractal time: 1/f-like power spectrum scaling of the human electroencephalogram. Int. J. Neurosci..

[13] Feinberg I, March JD, Floyd TC, Fein G, Aminoff MJ (1984). Log amplitude is a linear function of log frequency in NREM sleep EEG of young and elderly normal subjects. Electroencephalogr. Clin. Neurophysiol..

[14] Bódizs R (2021). A set of composite, non-redundant EEG measures of NREM sleep based on the power law scaling of the Fourier spectrum. Sci. Rep..

[15] Donoghue T (2020). Parameterizing neural power spectra into periodic and aperiodic components. Nat. Neurosci..

[16] Colombo MA (2019). The spectral exponent of the resting EEG indexes the presence of consciousness during unresponsiveness induced by propofol, xenon, and ketamine. Neuroimage.

[17] Lendner JD (2020). An electrophysiological marker of arousal level in humans. Elife.

[18] Miskovic V, MacDonald KJ, Rhodes LJ, Cote KA (2019). Changes in EEG multiscale entropy and power-law frequency scaling during the human sleep cycle. Hum. Brain Mapp..

[19] Freeman WJ, Holmes MD, West GA, Vanhatalo S (2006). Fine spatiotemporal structure of phase in human intracranial EEG. Clin. Neurophysiol..

[20] Gao R, Peterson EJ, Voytek B (2017). Inferring synaptic excitation/inhibition balance from field potentials. Neuroimage.

[21] Webb WB, Agnew HW (1971). Stage 4 sleep: Influence of time course variables. Science.

[22] Tucker AM, Dinges DF, Van Dongen HPA (2007). Trait interindividual differences in the sleep physiology of healthy young adults. J. Sleep Res..

[23] Hertenstein E (2018). Reference data for polysomnography-measured and subjective sleep in healthy adults. J. Clin. Sleep Med..

[24] Gander P, Signal L, Van Dongen HPA, Muller D, Van Den Berg M (2010). Stable inter-individual differences in slow-wave sleep during nocturnal sleep and naps. Sleep Biol. Rhythms.

[25] Kullback S, Leibler RA (1951). On Information and Sufficiency. Ann. Math. Stat..

[26] Taillard J, Gronfier C, Bioulac S, Philip P, Sagaspe P (2021). Sleep in normal aging, homeostatic and circadian regulation and vulnerability to sleep deprivation. Brain Sci..

[27] Carrier J, Land S, Buysse DJ, Kupfer DJ, Monk TH (2001). The effects of age and gender on sleep EEG power spectral density in the middle years of life (ages 20–60 years old). Psychophysiology.

[28] Landolt HP, Dijk DJ, Achermann P, Borbély AA (1996). Effect of age on the sleep EEG: Slow-wave activity and spindle frequency activity in young and middle-aged men. Brain Res..

[29] Dijk DJ, Beersma DGM, van den Hoofdakker RH (1989). All night spectral analysis of EEG sleep in young adult and middle-aged male subjects. Neurobiol. Aging.

[30] Werth E, Achermann P, Borbély AA (1997). Brain topography of the human sleep EEG: Antero-posterior shifts of spectral power. NeuroReport.

[31] Andrillon T (2020). Revisiting the value of polysomnographic data in insomnia: More than meets the eye. Sleep Med..

[32] Weber J, Klein T, Abeln V (2020). Shifts in broadband power and alpha peak frequency observed during long-term isolation. Sci. Rep..

[33] Markovic A, Kaess M, Tarokh L (2020). Gender differences in adolescent sleep neurophysiology: A high-density sleep EEG study. Sci. Rep..

[34] Ujma PP (2014). Sleep spindles and intelligence: Evidence for a sexual Dimorphism. J. Neurosci..

[35] Campbell IG, Feinberg I (2016). Maturational patterns of sigma frequency power across childhood and adolescence: A longitudinal study. Sleep.

[36] Ujma PP, Sándor P, Szakadát S, Gombos F, Bódizs R (2016). Sleep spindles and intelligence in early childhood-developmental and trait-dependent aspects. Dev. Psychol..

[37] Werth E, Achermann P, Dijk DJ, Borbély AA (1997). Spindle frequency activity in the sleep EEG: Individual differences and topographic distribution. Electroencephalogr. Clin. Neurophysiol..

[38] Bódizs R, Körmendi J, Rigó P, Lázár AS (2009). The individual adjustment method of sleep spindle analysis: Methodological improvements and roots in the fingerprint paradigm. J. Neurosci. Methods.

[39] Wen H, Liu Z (2016). Separating fractal and oscillatory components in the power spectrum of neurophysiological signal. Brain Topogr..

[40] Bódizs R (2017). The hemispheric lateralization of sleep spindles in humans. Sleep Spindl. Cortical Up States.

