# Supplementary material for: Overnight dynamics in scale-free and oscillatory spectral parameters of NREM sleep EEG
Source: Sci Rep. 2022 Nov 1;12:18409. doi: 10.1038/s41598-022-23033-y (PMC9626458; doi:10.1038/s41598-022-23033-y)
Supplement: Supplementary file 1 — Supplementary Tables. [file 41598_2022_23033_MOESM1_ESM.pdf]

Overnight dynamics in scale-free and oscillatory spectral parameters of NREM sleep EEG

Csenge G. Horváth [1*], Orsolya Szalárdy [1,2], Péter P. Ujma [1], Péter Simor [3,4], Ferenc Gombos [5,6], Ilona Kovács[6], Martin Dresler[7], Róbert Bódizs [1]

[1]Institute of Behavioural Sciences, Semmelweis University, Budapest, Hungary

[2] Institute of Cognitive Neuroscience and Psychology, Research Centre for Natural Sciences, Budapest, Hungary

[3]Institute of Psychology, ELTE, Eötvös Loránd University, Budapest, Hungary

[4]UR2NF, Neuropsychology and Functional Neuroimaging Research Unit at CRCN – Center for Research in Cognition and Neurosciences and UNI – ULB Neurosciences Institute, Université Libre de Bruxelles (ULB), Brussels, Belgium

[5]Laboratory for Psychological Research, Pázmány Péter Catholic University, Budapest, Hungary

[6]ELRN-ELTE-PPKE Adolescent Development Research Group, Eötvös Loránd University, Budapest, Hungary

[7]Donders Institute for Brain, Cognition and Behaviour, Radboud University Medical Center, Nijmegen, The Netherlands

Suppl. Table 1. Percentage distribution of the location of maximal frequency shifts in the sample. Meaning of markings are the following: F-Fp: frontal-to-frontopolar, C-F: central-to-frontal, P-C: parietal-to-central, O-P: occipital-parietal. Numbers mean the percent of the occurrence of the maximum frequency shift in a given area.

	1. cycle (%)	2. cycle (%)	3. cycle (%)	4. cycle (%)
F-Fp	17.53	31.47	45.02	38.25
C-F	54.18	50.60	41.83	40.64
P-C	15.54	5.98	4.78	8.76
O-P	3.98	4.38	2.79	4.78

Suppl. Table 2. Means and standard deviations of $f_{maxPeak}$ values in different age groups on different regions. **Markings are the following: M-mean, SD-standard deviation, ch=children, t=teenager, y=young adult, m=middle-aged adult, Fp-Frontopolar region, F-Frontal region, C-central region, P-parietal region, O-Occipital region**

Region	M_{ch} (N=9)	SD	M_t (N=33)	SD	M_y (N=125)	SD	M_m (N=22)	SD
Fp	11.44	0.25	12.25	0.12	11.98	0.06	11.56	0.15
F	11.63	0.27	12.72	0.13	12.39	0.07	11.98	0.17
C	11.99	0.22	13.16	0.11	13.32	0.06	13.36	0.14
P	12.20	0.20	13.28	0.10	13.47	0.05	13.76	0.12
O	12.34	0.18	13.34	0.09	13.40	0.05	13.82	0.11

Suppl. Table 3. P values of post hoc test about the region x age group interactions with respect to the $f_{maxPeak}$ values. **Markings: ch=children, t=teenagers, y=young adults, m=middle-aged adults, Fp-frontopolar region, F-frontal region, C-central region, P-parietal region, O-occipital region**

Region	y vs. ch	y vs. m	y vs. t	ch vs. m	ch vs. t	m vs. t
Fp	0.995	0.809	0.978	1.000	0.672	0.044
F	0.796	0.769	0.805	1.000	0.090	0.008
C	0.002	1.000	1.000	0.002	0.016	1.000
P	0.003	0.999	1.000	0.000	0.029	0.719
O	0.021	0.879	1.000	0.000	0.046	0.658
